# What Drives the Performance of Collaboration Networks: A Qualitative Comparative Analysis of Local Water Governance in China

**DOI:** 10.3390/ijerph17061819

**Published:** 2020-03-11

**Authors:** Can Cui, Hongtao Yi

**Affiliations:** 1 Department of Politics and Public Administration, The University of Hong Kong, Pokfulam, Hong Kong, China; cui0601@hku.hk; 2 School of Public Administration and Policy, Renmin University of China, Beijing 100872, China; 3 John Glenn College of Public Affairs, The Ohio State University, Columbus, OH 43210, USA

**Keywords:** collaboration networks, social capital, network performance, water governance, fsQCA

## Abstract

Local water governance is challenging given the significance to public health and the difficulties to manage it in a fragmented administrative system. A collaboration network is a popular governance tool in local governance to cope with functional fragmentation problems and institutional collective action (ICA) dilemmas. Empirical works are needed to examine the outcomes of such governance networks, especially in the context of environmental governance. With fuzzy-set qualitative comparative analysis (fsQCA), this article seeks to evaluate the outcomes of collaboration networks by investigating the influence of network structures on local water governance performance in China. Based on empirical analyses on a dataset of twenty local water governance networks implementing the Water Ecological Civilization Pilot Project in China, the findings suggest that a high overall bridging and bonding of social capital and a low network density are important determinants of effective collaboration networks. This study has policy implications for the design of local collaboration networks in facilitating effective environmental governance.

## 1. Introduction

Fragmentation among government units and across jurisdictions poses serious challenges to local governments in their collective efforts to promote effective regional governance [1]. Such challenges are termed as institutional collective action (ICA) dilemmas [2]. One of the mechanisms adopted to alleviate these dilemmas is for policy actors to form collaboration networks. These networks become effective governance tools to deliver public services and implement government policies through reducing functional and horizontal fragmentation by alleviating the information cost, bargaining cost and enforcement cost incurred during policy implementation [1]. However, very few studies have empirically examined the network-level performance of collaboration networks, especially in the context of China.

Performance evaluation has always been a focal question in the literature of public policy and management [3,4,5]. In the context of network governance, network performance represents a significant stream of research in public administration [6,7,8]. With the recent advancement in data collection and analysis methodologies in network studies [9], scholars have pushed the boundaries of the performance evaluation of collaboration networks both theoretically and methodologically [10,11,12].

We examine what factors motivate better network outcomes in the context of water governance collaboration networks in China. Water management always poses multi-dimensional challenges to governance [13,14]. The presence of collaboration networks in water management enables public managers to overcome traditional governance challenges, such as functional fragmentation. Focusing on the twenty cases of the Water Ecological Civilization Pilot Project implemented since 2013, this study evaluates the effectiveness of these local collaboration networks informed by social capital theory and network performance research. We hypothesize that bonding social capital, bridging social capital and network density are important causal conditions for desirable performance of water governance networks. Different from regression-based large-N studies, this article employs fuzzy-set qualitative comparative analysis (fsQCA) to analyze the complex causal configurations behind the network outcomes. fsQCA helps detect set relations and reveal more details from cases. It is meaningful to open the “black box” of local water governance with consideration of the complex components of collaboration networks. The results of the fsQCA analysis suggest three different configurations that could lead to desirable network performance, addressing the significance of local conditions and social capital embedded in the networks. The first causal path indicates the effect of low density and local treatment pressure on network performance, while the second one shows that the combination of high bridging capital, small secondary industry share and sufficient investment and pressure can facilitate the positive outcome. The third one speaks to the joint effect of bridging capital, bonding capital and adequate local attention. The study of network performance is theoretically essential to understand the role of social capital in collaboration networks. It also provides public managers with practical insights on network design and administration.

The rest of the paper is structured as follows. The following section presents the theoretical framework based on which we evaluate the performance of collaboration networks, linking social capital theory with network structures and network performance. We then introduce the background of the local water governance system in China, with details of the Water Ecological Civilization Pilot Project. Hypotheses are then proposed based on social capital theories and network governance research. With twenty prefecture-level water governance networks in China, fsQCA is utilized to test the hypotheses. The results of the fsQCA are then summarized and discussed. The article concludes with theoretical and practical implications, as well as its limitations.

## 2. Network Governance and Network Performance

### 2.1. Whole Network Performance

Provan and Milward [15] proposed a benchmark network effectiveness theory by emphasizing the role of integration, external control, stability and resources munificence in shaping the performance of mental health delivery networks in four big cities in the United States. Provan and Sebastian [16] further explained network effectiveness with clique analysis, showing the influence of integration on a network’s effectiveness. Provan and Milward [4] identified three different levels of analysis on network outcomes: community outcomes, network outcomes and organizational outcomes. Each level of analysis interacts with each other but is not necessarily interdependent [4]. Compared to the network effectiveness at the community and organization levels, network-level performance receives more attention. The network-level performance refers to positive outcomes achieved through the joint actions of participants in the network. In this study, it refers to the attainment of policy goals through cooperation in the collaboration networks. Contingency variables, such as trust, size of the network, goal consensus and task complexity, were found to affect network performance at the network level [5,17,18]. The strength and types of the ties between network actors, known as multiplexity, could also be an important predictor for network effectiveness [4,19].

Methodologically, extant literature evaluates network performance mainly with case studies [15,16]. Some studies conducted to evaluate the network performance in both private and public sectors employ panel data analysis [20,21,22], but their operationalization of networks are very simple, typically with dummy variables representing the presence of network relationships. As most studies on network performance apply either large-N studies [21] or case studies [15], evidence from a different methodological perspective, for example, small-N studies on network performance, is much needed [8]. This study contributes to the literature on small-N analysis of network performance, with fsQCA.

### 2.2. Forms of Network and the Influence of Social Capital on Performance

Various forms of a network exist in the practice of network governance. Depending on who manages the whole network, the network could be jointly governed, governed by a central organization, or by an external actor [5,23,24]. Alternatively, networks could also be categorized based on the mechanisms of their formation. Mandated networks are created by a government agency, and voluntary networks are created from a bottom-up agreement [23,25]. Self-organizing networks are decentralized and dynamic, whereas managed networks involve power and control from network managers [26,27]. With consideration of different characteristics of multiple networks, the water collaboration network in this study can be best described as a managed network with the Water Affairs Bureau (WAB) as a broker or lead organization in the network [5,28]. The performance of different types of networks is influenced by different factors. The drivers of performance in managed networks are not necessarily the same as self-organizing ones. Therefore, we are investigating the performance of twenty managed networks, different from extant studies that are mainly targeted at the performance of self-organizing networks [9,10].

Networks are a collection of social relations, which can be best understood with social capital theory. The notion of social capital indicates access to the connections and resources embedded in the network [29,30]. Social capital influences not only network structures, but also network outcomes [31,32,33]. Even though the concept of social capital is a bit confusing in the study of networks, most scholars agree with the positive impacts of social capital [29,34]. Different types of social capital have been discussed in the literature, including bridging social capital, bonding social capital and linking social capital [34]. Network scholars also made efforts to link bridging and bonding social capital with network structures [7,26]. For example, Berardo and Scholz [26] found that bridging structures and reciprocal bonding relationships function differently in resolving cooperation risk dilemmas and in managing networks. Yi [27] further examined the influence of bonding and bridging social capital on the performance of green energy policy networks in the U.S.

As for the structural variables of networks, Salancik [35] emphasized the importance of the structure of the interactions, including density, centralization and centrality, in achieving collective interests. Network size is another structural factor that is considered relevant for network performance [36]. The elements mentioned above are employed to explain the overall performance of the network as a whole rather than as individual organizations. For example, researchers examined the influence of centralization or density of the entire network on whole network performance [8,37]. However, mixed evidence is found regarding the effect of density [38]. 

Building upon these efforts, we further test the influence of social capital and network structures on network performance with two unique contributions. First, we examine the performance of designed networks, for which few studies have looked into the influence of social capital on network performance. Second, the networks under study are embedded in the context of China, where very few network studies have been conducted thus far.

### 2.3. Local Water Governance Networks in China 

Solving water-related challenges through effective water governance is always one of the priorities on China’s policy agenda. The water system was governed by a fragmented system of multiple agencies, lacking effective coordination among them. To address the fragmentation of authorities, local governments in China have started experimentation with reforms from fragmented systems to integrative water governance institutions [14]. In addition to structural reform of the water governance system, different collaborative practices have been adopted to overcome fragmentation challenges [6,39]. The Water Ecological Civilization Pilot Project is a compelling example of the government’s efforts to promote water sustainability by establishing collaboration networks among different local agencies. 

The National Water Ecological Civilization Pilot Project aims to address policy issues surrounding water supply, flooding and ecological conservation. Selected pilot cities were empowered to develop their own implementation plans considering their local conditions. However, the fundamental concepts of the experimental programs and the selection of the pilot cities are determined by the central government, rather than through a bottom-up process. The Chinese central government takes the responsibility to review local pilot program plans and conduct evaluations of the selected cities after the implementation. Key policy measures of the local pilot plans include water security, water ecology, water environment, water saving, water management and water culture. 

The first batch of the Water Ecological Civilization Pilot Project was launched in 2013 with the following objectives: to implement strict water management; optimize water configuration; strengthen the management of water saving; reinforce water conservation; promote protection and restoration of the ecosystem; and enhance awareness. Seventy-seven of 287 prefecture-level cities in China were selected as the 1st batch and the 2nd batch pilot cities in 2013 and 2014 to implement this innovative water governance program. Similar to urban housing reform in China, policymakers did not wait until a satisfactory performance was observed to extend the program to a broader scale [40]. Therefore, the performance of such a pilot project is still underexamined. 

Different from other projects, the Water Ecological Civilization Pilot Project mandated that departments to work together to address water-related challenges, which is an issue-focused collaboration network. Even though some cities have already passed the evaluation conducted by the central government, the evaluation of the overall performance of these collaboration networks should be conducted in a systematic way. 

## 3. Hypotheses

### 3.1. Bonding Social Capital and Water Governance Performance

Both the institutional collective action framework (ICA) and the ecology of games framework (EGF) emphasized the influence of social capital on collective outcomes in fragmented policy arenas [11,41]. Social capital, including bonding social capital and bridging social capital, has long been believed to help resolve cooperation and coordination problems [42]. Bonding social capital occurs when actors in a group create connections that bring them closer to each other, demonstrated by intensive overlapping ties, reciprocity and clustered transitive relationships [42]. The prevalence of bonding capital facilitates trust in network relationships, simulating trustworthy behaviors among policy actors [26,43]. Based on the trust and norms that the actors conducted, strong relationships in the network offer reliable communication channels that support long-term cooperation relationships [32,44]. More importantly, reciprocal bonding relationships are preferred to account for the risk of defection [26]. Bonding social capital embedded in the network enhances the overall capacity to detect and punish defection behaviors by individual actors [45]. Given the advantages in facilitating trust building and reducing cooperation risks, bonding social capital is anticipated to positively influence the effectiveness of the network. 

Bonding social capital indicated by a close-knit configuration in the network could be captured with the average clustering coefficient [7]. It is because bonding capital is generally represented by the presence of triangles where three members are linked. A larger percentage of triangles means a higher level of clustering in the network, and thus more bonding capital [42]. The average clustering coefficient measures the closeness among actors at the network level. The value of the average clustering coefficient reflects the extent of how well actors are linked with each other in the network [27,42]. We expect a positive effect of the average clustering coefficient on the performance of water governance networks.

**Bonding** **Social** **Capital** **Hypothesis** **(H1):**  *High bonding capital measured by the average clustering coefficient of the overall water governance network is one of the causal conditions of desirable water governance performance.*

### 3.2. Bridging Social Capital and Water Governance Performance

Bridging social capital is generated when actors in a group create connections beyond a smaller circle of acquaintances and extend the scale of the network by reaching out to other unfamiliar groups [42]. Well connected with a broader set of actors means wider access to nonoverlapping information, enhancing the capacity and resources of the actors. A bridging structure tackles coordination challenges through centralized brokers who sit in the center of the network, to coordinate various activities throughout the whole network [42]. Brokers fulfill their coordination roles by organizing centralized activities and serving as communication channels [27]. Although the existence of different groups may easily lead to fragmentation within the network, central actors are able to connect disjointed participants and facilitate innovative responses to problems, thus influencing the network outcomes [42,46]. Central actors typically require fewer links to reach others in the network, resulting in efficient information transmission [7]. Therefore, establishing smooth information channels and competent coordination are essential to generate effective collaborative outcomes [5], leading to the next hypothesis.

The term average degree is the mean of the connections that each node shares with others [7]. The average degree of centrality of the collaboration network indicates the overall degree of coordination in the collaboration network. The degree of coordination is reflected by a central organization that worked as a broker to access resources and serve as a coordinator in an interorganizational network. Degree centrality measures the extent to which an actor is a broker, which captures the network-level bridging social capital. A higher average degree of centrality reflects more centralized networks with greater inter-node connectivity and thus a higher level of bridging social capital embedded in the collaboration networks.

**Bridging** **Social** **Capital** **Hypothesis** **(H2):**  *High bridging social capital measured by the average degree centrality of the overall water governance network is one of the causal conditions of desirable water governance performance.*

### 3.3. Network Density and Water Governance Performance

Besides the average degree centrality and clustering coefficient, density is also an important indicator of network closure according to social capital theory. Density, one of the properties of the network, reflects a general level of linkages in the whole network. It is calculated by dividing the actual connections within a network over the maximum number of potential connections [36,47]. A density score of “1” means the organizations in the network are all connected to one another. 

However, mixed findings have been reported on the effect of density on network performance. Based on Coleman’s arguments on closure, Burt [32] contended that the density of the network is a source of social capital [31,32,45]. A higher density score indicates a higher level of closure, which leads to a more effective network. Studies analyzing how density affects the network outcomes also supported such an argument. For example, Wang [8] confirmed the positive effect of dense cooperation relationships on effective neighborhood governance networks through analyzing 22 community networks in Beijing. Yet, from the analysis of job performance, Burt [32] demonstrated that dense networks were associated with an unsatisfactory performance, supporting the structural hole theory. 

Our theoretical expectation on density takes both the theoretical background and the reality of the water governance system in China into account. Local water governance in China, historically, has been fragmented across multiple departments governing water-related functions. Through implementing the Water Ecological Civilization Pilot Project, local agencies started to form collaboration networks within their city boundaries. When the city government designs the network in a way that features multiple functions, and when many actors are involved in the same function, a high density is then observed. Therefore, if the network density is too high, it might signal functional fragmentation and oversubscription of responsibilities on agencies. Therefore, we expect low density to be related to better governance performance, as the sub-projects and responsibilities are jointly managed by a reasonable number of organizations in the governance network.

**Network** **Density** **Hypothesis** **(H3):**  *Low density of the overall water governance network is one of the causal conditions of desirable water governance performance.*

## 4. Methods and Data

### 4.1. Qualitative Comparative Analysis (QCA)

QCA is a comparative case-oriented research approach and a collection of techniques based on set theory and Boolean algebra [48,49]. According to set theory and Boolean algebra, a causal path consisting of the necessary and sufficient conditions can be identified without irrelevant conditions. Configuration analysis by QCA combines the advantages of both the qualitative method and quantitative method, particularly suitable to studies with a sample size between 10 and 100. With QCA, causal complexities are considered with special attention to the concepts of conjunctural causation, multifinality and equifinality [50,51]. Conjunctural causation means that different groups of factors may lead to the same result [52], while multifinality refers that the same factor could play different roles in different situations [53]. As a fundamental notion underlying QCA, equifinality assumes two or more causal configurations can be equally effective in stimulating high performance [54,55]. These concepts carried by QCA should be noted as compared to traditional statistical techniques.

QCA has been successfully applied to management and other social science subjects [8,56,57]. It is widely recognized by scholars with its three major techniques, namely crisp-set QCA (csQCA), multi-value QCA (mvQCA) and fuzzy-set QCA (fsQCA). Since the explanatory variables for network outcome cannot be simply defined as “0” (non-membership) or “1” (full membership), fsQCA best matches with the purpose of this study by calibrating the continuous values into interval-scale variables [51,58]. Calibration should be based on theoretical background and knowledge, or it should be based on substantive practical knowledge if theoretical knowledge is not available [58]. It is worth mentioning that cases in QCA can have different membership scores in a set. Depending on the membership scores, some concepts are crucial in the analytic process. Logic remainders refers to “the truth table rows for which not enough empirical evidence is at hand” [59] (p. 324). Another important concept in QCA is consistency, which indicates that the degree to which the empirical data are in accordance with the assumed subset relation. Consistency in QCA measures “the percentage of cases’ set-membership scores in two sets that is in line with the statement that one of the two sets in a subset (or superset) of the other” [59] (p. 329).

Following the abovementioned concepts of QCA, it is appropriate for this article to detect the driving forces of local water governance performance with the acknowledgment of set relations. Local water governance is always complicated, which cannot be explained by simple correlations. Set relations combining different conditions allow for comprehensive and intuitive analysis of the complex problem. It is a more direct and easy way for configuration determination. Moreover, it is difficult to collect large samples of non-public policy implementation plans of local governments. Due to the relatively small but the best available dataset, QCA is a good practice for small-N research, providing a deep understanding of cases beyond simple correlation. More importantly, the set membership scores, in this case, should not be crudely calibrated by “0′s” and “1′s”, as the structural components of networks are diversified to varying degrees. Therefore, this study applied fsQCA with the software fsqca3.0 for empirical analyses [60].

All possible combinations of causal conditions are collected in a truth table, and the QCA solutions are presented after manual editing based on the truth table. In our study, logical remainders are kept if they have raw consistency scores over 0.85, otherwise they are deleted [58,60]. In total, there are twenty water ecological governance networks included as cases in this research, which are all prefecture-level cities implementing the Water Ecological Civilization Pilot Project in China. Truth table for the analysis is presented in Table S1 in the Supplementary Materials.

### 4.2. Data

#### 4.2.1. Data Collection

**Figure 1:**
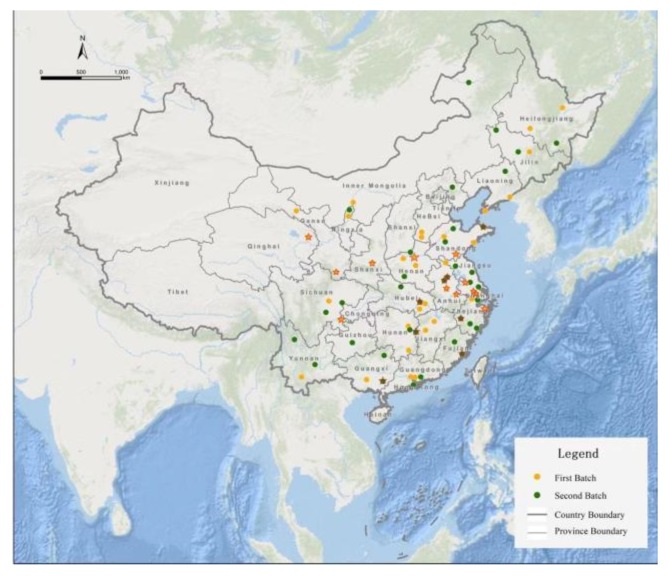
Distribution of the selected cities in China. The twenty selected cities are symbolized with a pentagram (★).

This study examined the performance of water governance pilot project by analyzing twenty collaboration networks that implemented the Water Ecological Civilization Pilot Project at the local level in China. In order to better understand the roles played and tasks undertaken by local governments in the implementation process, we collected implementation plans of the pilot project from twenty cities in China. Due to the difficulty in collecting such non-mandatory policy documents, we were only able to obtain a sample of twenty implementation plans. These twenty cities across thirteen different provinces are representative of the seventy-seven pilot cities because they are reasonably distributed in various areas in China and in two time points of implementing the Water Ecological Civilization Pilot Project. In addition, the twenty cities represent the complexity and diversity of local water governance in different degrees. Among the twenty cities, some of them are traditionally regarded as high-class ecological cities while some were recently reported as poorly or moderately performing cities regarding environmental governance. For example, Nanning in Guangxi province faced severe water governance challenges, especially in the governance of Nakao River. In the worst case, there are more than forty sewage outlets discharging sewage to the river. More seriously, garbage and construction spoil piled up to occupy the river, which brought extensive governance pressures to local governance [61]. However, Wuxi in Jiangsu province who used to be the outbreak city of blue algae has performed well in improving its water environment in recent years. The quality of Taihu Lake generally reached Class IV and thirty-eight black and odorous water bodies have been rectified [62]. The twenty cities basically cover different cities with different water governance problems and varying governance performance. Therefore, it is reasonable to believe that the twenty cases represent a relatively comprehensive picture of the population who implemented the Water Ecological Civilization Pilot Project. Figure 1 visualizes the distribution of the pilot cities and the selected cases.

**Figure 2:**
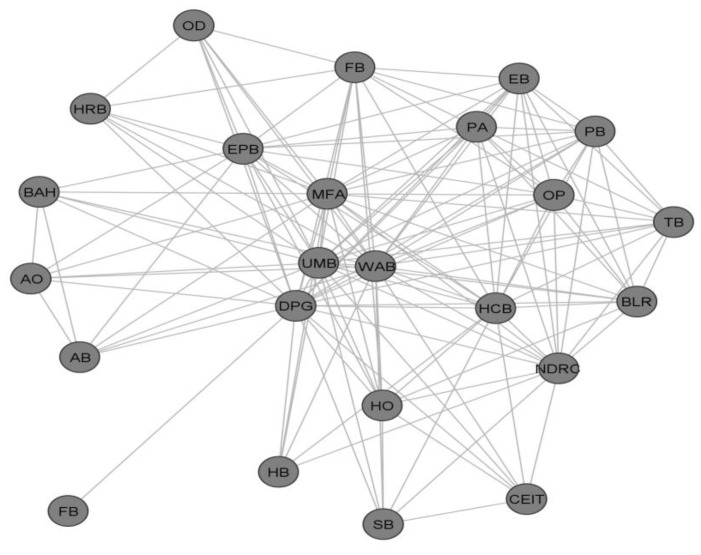
Visualization of the water governance collaboration network in Yantai. *WAB: Water Affairs Bureau; UMB: Urban Management Bureau; MFA: Marine and Fisheries Agency; OD: Organization Department; HRB: Human Resources and Social Security Department; BAH: Bureau of Animal Husbandry; AO: Office of Agriculture and Industry; AB: Agricultural Bureau; FB: Finance Bureau; EPB: Environmental Protection Bureau; MFB: Municipal Forestry Bureau; DPG: District People’s Government; HB: Health Bureau; HO: Hydrographic Office; SB: Statistics Bureau; EB: Education Bureau; PA: Agency of Planning; PB: Bureau of Culture, Radio, Film, TV, Press and Publication; OP: Propaganda Department; TB: Tourism Bureau; BLR: Bureau of Land Resources; HCB: Housing Construction Bureau; CEIT: Commission of Economy and Information Technology. NDRC: National Development and Reform Commission.

Some of the implementation plans were directly downloaded from the official website of city governments and the website of the Water Affairs Bureau; the rest were collected by reaching out to local officials via social media (WeChat) or emails. After we received implementation plans from the twenty cities, we manually coded the functions and agencies according to the division of duties in the implementation plans. Through cleaning the information of the responsible departments, we obtained a two-mode network of functions and agencies. Functions may include wastewater treatment, water quality, water recycled and reused, water culture publicity and water-related education etc. The corresponding agencies are Water Affairs Bureau, Urban Management Bureau, Organization Department, and Education Bureau, etc. Then we transformed the two-mode network data into a one-mode network to capture the collaboration network among agencies with UCINET. The transformation from a two-mode collaboration network to a one-mode collaboration network enables us to observe a direct relationship between the involved departments. Figure 2 presents the collaboration network with nodes representing the actors involved (municipal government departments) and ties representing the interactions (i.e., being assigned to the same function) between departments in the City of Yantai, one of the pilot cities implementing the Water Ecological Civilization Pilot Project in the Shandong province.

The collaboration network in Yantai represents one of the twenty collaboration networks that we analyzed. Although the number of actors in these networks are not exactly the same, the collaboration network presented in Figure 2 still provides us with a relatively representative picture of how departments interact with each other through carrying out the water-related functions. We then calculate the average clustering coefficient, network density and average degree centrality using UCINET. These data allow us to evaluate how these network structural features are related to governance performance, as we compare across the twenty cities.

#### 4.2.2. Network Performance: Wastewater Treatment Capacity 

To evaluate the Water Ecological Civilization Pilot Project, the outcome of the water governance network can be assessed by evaluating the accomplishment of the project objective. Through analyzing local implementation plans collected by the authors, wastewater treatment is one of the most crucial tasks of the Water Ecological Civilization Pilot Project. Although this project has multiple objectives, wastewater treatment is considered as the top priority among numerous tasks from the analysis of the financial budget allocated to each item, including wastewater treatment, flood control, water culture, water management and water safety. It is understandable because wastewater treatment is closely related to water pollution problems that have reached a record level in China. The capacity of wastewater treatment could efficiently alleviate environmental pressure resulting from water pollution and water scarcity [63].

Based on the practical and theoretical background, this article measured the performance of the water governance network using wastewater treatment capacity collected from the China City Construction Yearbook in 2015, the latest data available. Referring to the direct calibration method, we chose three important anchors: threshold for full membership, the crossover point and the threshold for full non-membership. For the water governance outcome, it was calibrated to the 30th percentile, 50th percentile and 70th percentile, respectively. 

#### 4.2.3. Causal Conditions

Following our hypotheses, we calculated the average degree centrality, average clustering coefficient and density score of the water governance networks with UCINET, and then calibrated the raw data from zero to one, which was then analyzed in fsQCA software. The network-related variables were calibrated based on our substantive knowledge of network variables. For example, a network with a density score over 0.7 could be regarded as a dense network, where 0.7 is the threshold for full membership in a set.

Besides the structural variables of collaboration networks, we also included variables related to water governance, namely the percentage of secondary industry in a city, investment on fixed assets and the quantity of wastewater discharged. Those variables could capture the local conditions related to water governance. The percentage of secondary industry in a city measures the city’s level of industrialization while investment in fixed assets tells about local attention on infrastructure construction. The quantity of wastewater discharged directly reflects the cities’ treatment pressure. 

All these variables were calibrated to the 30th percentile, 50th percentile and 70th percentile. The data on network performance, attributes of the city and water-relevant data were all collected from the China City Statistical Yearbook and China City Construction Statistical Yearbook in 2015. Table 1 reports data sources, measurement and calibration of the aforementioned variables. Table 2 summarizes the descriptive statistics of these variables.

## 5. Results

Table 3 reports the intermediate solution for the network outcome. Assumptions underlying the standard analysis are summarized in Table S2 in the Supplementary Materials. Intermediate solutions were adopted in this study, because it incorporates logical remainders that correspond with theoretical assumptions and practical knowledge. The complex solution uses no logical remainders, whereas a parsimonious solution includes all logical remainders without any evaluation of rationality. Given its comparable rationality, the intermediate solution is commonly employed by scholars to interpret the results [51,58,64,65]. Complex solution for the network outcome in this study is included in Table S3 in the Supplementary Materials for further reference.

Consistency and coverage are two critical components of the QCA results. “Consistency gauges the degree to which the cases sharing a given combination of conditions agree in displaying the outcome in question when coverage indicates how closely a perfect subset relation is approximated” [58] (p. 44). As argued by Ragin [66], the consistency cutoff should not be lower than 0.75, and the cutoff is recommended to set equal or higher than 0.8. In our study, we set the consistency cutoff to 0.85, a reasonably high value. Three causal paths in our results are reported with the notation system commonly used in QCA analysis [8,58]. 

The first causal path was the combination of low membership in the sets of density and high membership in the sets of the quantity of wastewater discharged. This suggests that water governance networks with a low density, together with a high quantity of wastewater discharged, are very likely to be effective in treating wastewater. Other conditions in the first causal path were irrelevant. Hence, when local policy practitioners form a less dense collaboration network to implement the Water Ecological Civilization Pilot Framework under heavy treatment pressure, the established collaboration network is more likely to obtain higher performance. Seven cases were covered by this causal path, as reported in Table 3. It is interesting to note that low density is one of the causal conditions of high performance, which is consistent with our third hypothesis.

The second causal path was the combination of high membership in the sets of the average degree centrality, investment in fixed assets, the quantity of wastewater discharged and low membership in the sets of the percentage of the secondary industry. This causal path supported our second hypothesis related to bridging social capital. It suggests that cities with high bridging capital, high investment and treatment pressure, as well as a low secondary industry share, are more likely to achieve desirable water governance performance. Five cases are consistent with this configuration. The third causal path was the combination of high membership in the sets of the average degree centrality, clustering coefficient, the quantity of wastewater discharged and investment in fixed assets. In other words, a city is very likely to achieve a high wastewater treatment capacity if the city has a water collaboration network with high bridging and bonding social capital and has a large quantity of wastewater discharged and heavy investment in fixed assets, which supported our first and second hypotheses. Five cases demonstrated this configuration. Robustness checks using different consistency cutoffs and crossover points are introduced in detail in Table S4 and Table S5 in the Supplementary Materials.

## 6. Discussions

The fsQCA analysis identified three different causal paths for the presence of wastewater treatment capacity. To better understand the results, we should keep in mind that Boolean methods behind QCA represents each case with a combination of causal and outcome conditions [48]. The first causal path identified the effect of network density, along with the quantity of wastewater discharged, on the performance of water governance networks. Different from the findings of neighborhood governance networks in Beijing, arguing that a high network density is an important component of network effectiveness [8], we find that low membership in the sets of density, combined with a large quantity of wastewater discharged, leads to positive water governance performance in our study. A large quantity of wastewater discharged, including the quantity of industrial wastewater and residential wastewater, indicates heavy pressures to treat the wastewater. However, this result does not necessarily mean less connected networks are more likely to perform well. Density in water governance projects should be considered in the context of the water governance system. Low density, as one component of the first causal path, means that the assigned functions in pilot projects are either too few to be distributed among network participants or are covered by several vital actors. It signals a reasonable subscription of water-relevant responsibilities to local agencies. Low density also can be explained by the same functions being covered by a limited number of key actors, and thus is related to low levels of fragmentation in the network. Overall, this causal path means that collaboration networks with less fragmentation and greater treatment pressure seem more effective in treating wastewater, leading to positive water governance performance. For example, Hefei formed a less dense collaboration network that assigns similar responsibilities to several key actors, namely the Water Affairs Bureau, Environmental Protection Bureau and Construction Bureau, ensuring accountability and a coordination mechanism. At the same time, Hefei faces severe water governance challenges, especially wastewater collection and treatment. In this sense, under a heavy burden to enhance water governance efficiency, the relatively sparse network that distributes responsibilities reasonably could facilitate the positive outcome.

The second and the third causal paths identified by fsQCA confirmed our hypotheses on social capital. Bridging social capital measured by the average degree centrality, along with three other environmental factors, including the quantity of wastewater discharged, investment on fixed assets, and the low percentage of the secondary industry to local GDP, leads to effective water governance. The second causal path indicates that collaborative networks with high bridging social capital with heavy treatment pressure and sufficient investment in infrastructure in less-industrial cities facilitate the desirable water governance performance. It suggests that the structure of the collaboration networks and the local conditions of the industrial structure and water environment affect governance performance. High bridging capital embedded in the collaboration networks could resolve the cooperation dilemmas through brokers. Communication channels established through bridging capital in the networks could greatly help improve effectiveness. Along with sufficient attention and industrial structure, the network should perform reasonably well.

Bonding capital, indicated by the average clustering coefficient, together with high bridging capital and high memberships in two other environmental factors, leads to high performance in water governance. The last causal path further points out the combining effect of the components of collaboration networks and the variables of local conditions. High bonding and bridging capital, along with heavy treatment pressure and sufficient relevant investment, can advance water governance performance. High bonding capital can bring participants closer and simulate trust, and therefore reduce the cooperation risks and facilitate the positive outcomes. The effect of bonding capital becomes even stronger when it appears together with bridging capital and sufficient local governance attention and pressure. Local conditions are very important for the achievement of governance performance; but, if the city could formulate the collaboration network with adequate bridging and bonding capital, ensuring effective information flow and low cooperation risk, the city is still able to achieve positive governance outcomes. The major causal conditions, namely density, bridging capital and bonding capital, do not necessarily occur in each causal path, but their occurrence suggests the significance of the structural components of collaboration networks in shaping performance. The condition of the amount of wastewater discharged appeared in all three causal paths, which is a key causal condition. It is not difficult to understand that a high treatment pressure with a large amount of investment could facilitate decent treatment capacity. The presence of the average clustering coefficient and average degree centrality together further confirmed the importance of social capital and its significance in explaining network effectiveness.

## 7. Conclusions

Drawing on social capital theory and network performance research, this study aims to evaluate the network-level performance of local water governance in China. With the implementation of the Water Ecological Civilization Pilot Project in China and twenty cases of pilot cities, we are able to test the influence of bonding and bridging social capital on network performance with fsQCA. The fsQCA results revealed that high membership in the sets of the average degree centrality and clustering coefficient lead to high performance in water governance networks, indicating the significance of social capital in network governance. Network actors should pay attention to social capital embedded in their networks in order to achieve common goals and enhance overall performance. This study confirms the findings from social capital literature and network effectiveness literature on the positive influence of bonding and bridging social capital. At the same time, this study also finds that the impact of low network density is related to effective networks, motivating scholars to reconsider the effect of the structural variables of the network in a broader picture in terms of network design, cooperation and division of labor. More importantly, the results suggest that there are no simple prescriptions for achieving positive network outcomes, based on the fact that we find complex causal relationships behind the desirable performance.

We made a connection between network analysis and social capital theory in the context of China, which traditionally features a fragmented water governance system. Scholars are struggling with the difficulty in obtaining high-quality data for examining network effectiveness. The inspiration from small-N studies and complex causal configurations allows us to examine network effectiveness that is otherwise impossible to do. This study also provides public managers with practical insights on network design and collaboration process. Practitioners should recognize the influence and power of central actors in the network in coordinating with other network participants, as well as the significance of bonding structure in building trust and reducing cooperation risks. 

This article has several limitations that we need to acknowledge. First, due to data availability, this research only adopts one outcome variable, although it is one of the most important indicators of the project. Future studies could utilize more outcome variables, should they become available, to confirm the results. Moreover, the dynamic evaluation of the effectiveness might present a more comprehensive picture of the network performance. We also acknowledged that the samples in this study include both first-round and second-round pilot cities, which could potentially ignore the learning effect in the analysis of policy outcomes. Further studies could conduct a comparative analysis to account for the effect. Meanwhile, including more cases that are not self-motivated to apply for the pilot scheme can greatly enhance the generalizability of the results, which can also be the direction of future studies.

## Figures and Tables

**Table 1:** Variables, sources and calibration.

Variables	Description	Data Sources	Calibration
*Outcome*			
Wastewater Treatment Capacity	The quantity of wastewater been treated per day (unit: 10,000 m^3^/day)	China City Construction Statistical Yearbook	Fully in ≥ 103 Crossover = 51 Fully out ≤ 25
*Conditions*			
Degree Centrality	Bridging social capital of water governance network	Calculated with UCINET	Fully in ≥ 21 Crossover = 8 Fully out ≤ 4.5
Clustering Coefficient	Bonding social capital of water governance network	Calculated with UCINET	Fully in ≥ 3.99 Crossover = 1.95 Fully out ≤ 1.41
Density	Functional fragmentation of water governance network	Calculated with UCINET	Fully in ≥ 0.7 Crossover = 0.5 Fully out ≤ 0.3
Fixed-asset Investment	The completed investment on fixed assets in 2015 (unit: 10,000 Yuan)	China City Construction Statistical Yearbook	Fully in ≥ 1792685.7 Crossover = 520200.5 Fully out ≤ 373493.2
Secondary Industry as Percentage to GRP	The secondary industry as percentage to Gross Regional Product	China City Statistical Yearbook	Fully in ≥ 52% Crossover = 48% Fully out ≤ 43%
Quantity of Wastewater Discharged	The quantity of wastewater had been discharged in 2015 (unit: 10,000 m^3)^	China City Construction Statistical Yearbook	Fully in ≥ 39,032 Crossover = 15264.5 Fully out ≤ 7640

**Table 2:** Descriptive Statistics.

Variables	Observations	Mean	Median	Min	Max
Wastewater Treatment Capacity	20	76.43	72.06	1.5	256.4
Degree Centrality	20	12.9	7.46	1.25	45.82
Clustering Coefficient	20	3.52	4.24	0	19.44
Density	20	0.33	0.2	0.107	0.91
Fixed-asset Investment	20	1,405,277	2,023,452	19,286	8,861,396
Secondary Industry Share	20	47.67	11.4	14.33	65.68
Quantity of Wastewater Discharged	20	26,209.6	23,984.9	348	83,243

**Table 3:** Intermediate solution for the positive outcome (treatment capacity).

Configurations	Causal Paths		
Degree Centrality		●	●
Clustering Coefficient			●
Density	⊗		
Wastewater Discharged	●	●	●
Secondary Industry Share		⊗	
Fixed-Asset Investment		●	●
Raw Coverage	0.71	0.4	0.47
Unique Coverage	0.42	0.05	0.01
Consistency	0.93	1	0.99
Cases Covered	Ningbo; Hefei; Nanning; Wuxi; Suzhou; Wuhan; Linyi	Xi’an; Wuhan; Nanning; Zhengzhou; Suzhou	Suzhou; Zhengzhou; Wuhan; Hefei; Yantai
Overall Solution Coverage	0.95		
Overall Solution Consistency	0.95		

Note: Frequency cutoff = 1 and consistent cutoff = 0.85; multiple covered case: 4. A black circle (●) means a high membership in the condition and a circle with a cross-out (⊗) suggests a low membership. A blank in the causal path indicates that this condition is irrelevant in the results.

## Supplementary Materials

The following are available online at https://www.mdpi.com/1660-4601/17/6/1819/s1, Table S1: Truth table for the analysis of positive network performance, Table S2: Standard analysis assumptions, Table S3: Complex solution for the positive outcome, Table S4: Robustness check (consistency cutoff), Table S5: Robustness check (crossover point).
