# Supplementary material for: What Drives the Performance of Collaboration Networks: A Qualitative Comparative Analysis of Local Water Governance in China
Source: Int J Environ Res Public Health. 2020 Mar 11;17(6):1819. doi: 10.3390/ijerph17061819 (PMC7143648; doi:10.3390/ijerph17061819)
Supplement: Supplementary file 1 [file ijerph-17-01819-s001.pdf]

International Journal of
Environmental Research and Public Health

MDPI

Supplementary Material

1. Calibration

The membership scores for cases should be calculated by the calibration of sets either in crisp-set or in fuzzy-set QCA. Ragin (2008) suggested that the calibration should be based on theoretical or substantive knowledge, and substantive knowledge works as the major source when theoretical knowledge is unavailable. The direct calibration was employed in this study, with three cutoffs: the threshold for full membership in a set, the threshold for full non-membership, and the crossover point. As emphasized in the text, even though this article calibrates to percentiles, those variables are calibrated based on our substantive knowledge of network variables and investigation of the fact through interviews.

1. Analytic Process

1.1. Truth table

Table 1. Truth table for the analysis of positive network performance.

Conditions						Outcome	Number of Cases	Consistency
Clustering coefficient	Degree centrality	Density	Investment	Wastewater Discharged	Secondary industry share			
1	1	0	1	1	0	1	2	1
0	1	1	1	1	0	1	1	1
1	1	1	1	1	0	1	1	1
0	0	0	1	1	1	1	1	1
1	1	0	1	1	1	1	1	0.99
1	1	1	1	1	1	1	1	0.99
0	0	0	1	1	0	1	2	0.9
0	0	0	0	1	1	1	1	0.83
1	1	0	1	0	1	0	1	0.5
0	0	0	0	0	1	0	2	0.5
1	0	0	0	0	1	0	1	0.36
1	1	0	0	0	1	0	2	0.25
1	1	0	0	0	0	0	1	0.16
0	0	0	0	0	0	0	3	0.11

1.2. Assumption behind the analysis

Following theoretical support, the assumption of conditions is as followed,

Table 2. Standard analysis assumptions.

Conditions	Assumptions	Hypothesis
Clustering coefficient	Present	Hypothesis#1
Degree centrality	Present	Hypothesis#2
Density	Present/Absent	Hypothesis#3
Investment	Present	
Wastewater Discharged	Present	
Secondary Industry share	Absent	

1.3. Complex solutions

The complex solution is also reported here for reference. From the results, the intermediate solution makes more sense to this study not only in theory but also in practice.

Table 3. Complex solution for the positive outcome (Treatment Capacity).

Configurations	Causal Paths			
Degree Centrality	●	⊗	⊗	●
Clustering Coefficient	●	⊗	⊗	
Density		⊗	⊗	●
Wastewater Discharged	●	●	●	●
Secondary Industry Share		⊗		⊗
Fixed-asset Investment	●		●	●
Raw coverage	0.47	0.23	0.29	0.26
Unique coverage	0.24	0.1	0.15	0.05
Consistency	0.99	0.89	0.93	1
Cases Covered	Zhengzhou; Hefei; Wuhan; Suzhou; Yantai	Ningbo; Linyi	Ningbo; Nanning; Wuxi	Xi'an; Zhengzhou
Overall solution coverage	0.87			
Overall solution consistency	0.94			

2. Robustness Check

To check the robustness of the key findings, we conducted several robustness checks. First, we checked whether the results were robust to the alternative consistency cutoff. The minimum of consistency cutoff allowed in fsQCA is 0.75, and therefore we tried 0.8 and 0.9. The causal paths were exactly the same if the consistency cutoff were changed to 0.8. When the consistency cutoff changes to 0.9, the first causal path changed a little with fixed-asset investment as a relevant condition, as shown in Table S4. As the other two causal paths remained the same without any effects on our primary hypotheses, it is safe to conclude the results were pretty robust.

Table 4. Robustness check (consistency cutoff).

Configurations	Causal Paths		
Degree Centrality		●	●
Clustering Coefficient			●
Density	⊗		
Wastewater Discharged	●	●	●
Secondary Industry Share		⊗	
Fixed-asset Investment	●	●	●
Raw coverage	0.57	0.37	0.47
Unique coverage	0.27	0.05	0.01
Consistency	0.96	1	1
Cases Covered	Ningbo; Hefei; Nanning; Wuxi; Suzhou; Wuhan	Xi'an; Zhengzhou; Wuhan; Suzhou	Zhengzhou; Wuhan; Suzhou; Hefei; Yantai
Overall solution coverage		0.8	
Overall solution consistency		0.97	

Note: Frequency cutoff = 1, consistency cutoff=0.9; Multiple covered case: 3.

Later on, we checked whether the findings were robust to the alternative specification of the thresholds of the calibration. Following the practice of Wang (2016) and Fiss (2011), we changed two crossover points since the crossover point holds the maximum ambiguity to decide whether or not it is in the set. The change of the crossover point was mainly based on substantive knowledge, and the magnitude was within 30%. Then, we compared the new intermediate solutions with the original findings. The results were reported in the following Table S5.

Table 5. Robustness check (crossover point).

**Configurations**	**Robustness Check**						
	1st round				2nd round		
	Original crossover point	1st round crossover point	Causal path		2nd round crossover point	Causal path	
Degree Centrality	8	6.5			10		●
Clustering Coefficient	1.95	1.8	●		2.1		●
Density	0.5	0.4			0.6		⊗
Wastewater Discharged	15264.5	13000	●	●	17000	●	
Secondary Industry Share	0.48	0.45	⊗	⊗	0.5	⊗	⊗
Fixed-asset Investment	520200.5	450000		●	600000	●	●
Raw coverage			0.49			0.8	0.42
Unique coverage			0.15			0.51	0.13
Consistency			0.87			0.97	0.93
Overall solution coverage			0.97			0.94	
Overall solution consistency			0.93			0.97	

From Table S5, the results are generally robust to the adjustments of the crossover points. However, the causal path combining different attributes of local cities becomes more significant than the original result. We could understand the new solutions are the subsets of the original one, which support the argument that local conditions also matter to the performance. When we looked at the cases again, the crossover point made more sense at the original level. Also, in order to best understand the effect of network components on network effectiveness. Therefore, we decided to accept the configurations reported in the paper.

References

1. Fiss, P. C. Building better causal theories: A fuzzy set approach to typologies in organization research. *Academy of Manag. Journal*. **2011**,*54*, 393-420.
2. Ragin, C.C. *Redesigning Social Inquiry: Fuzzy Sets and Beyond*; University of Chicago Press: Chicago, IL, USA, 2008.
3. Wang, W. Exploring the determinants of network effectiveness: The case of neighborhood governance networks in Beijing. *Journal of Public Adm. Res. and Theory*. **2016**, *26*, 375-388.
